# Is fluoroscopy necessary during flexible ureteroscopy for the treatment of renal stones?

**DOI:** 10.1080/2090598X.2019.1702242

**Published:** 2019-12-18

**Authors:** Mustafa Kirac, Burak Kopru, Giray Ergin, Yusuf Kibar, Hasan Biri

**Affiliations:** Department of Urology, Koru Ankara Hospital, Yuksek Ihtisas University, Ankara, Turkey

**Keywords:** Fluoroless, radiation, renal stone, flexible ureteroscopy, stone treatment

## Abstract

**Objective:**

To investigate the feasibility and effectiveness of flexible ureteroscopy (fURS) without fluoroscopy during the treatment of renal stones.

**Patients and methods:**

Between April 2013 and August 2018, 744 patients’ data were evaluated retrospectively. Of these, 576 patients were included in the study. All fURS were performed by experienced surgeons. All procedures were planned with zero-dose fluoroscopy. But, if fluoroscopy was necessary for any reasons, these patients were excluded from the study. Demographic data, perioperative parameters, stone-free rate (SFR), and complication rates were recorded.

**Results:**

Of the patients planned for fluoroless fURS (ffURS), the procedure was successfully achieved in 96.7% (557/576 patients), as 19 patients required fluoroscopy during the procedure for various reasons. In the patients included in the study, the mean (SD) stone size was 11.6 (5.2) mm and the mean (SD) operating time was 39.4 (8.2) min. After the first session of ffURS, the SFR was 83.3% (achieved in 464 patients). Second and third sessions of ffURS were performed in 32 (5.7%) and seven (1.2%) patients, respectively. Overall, the complication rate was 11.8% and all complications were minor (Clavien–Dindo Grade I or II).

**Conclusions:**

The ffURS technique seems to be a safe and effective treatment compared to conventional fURS in patients with renal stones. This procedure should be performed in experienced centers, where fluoroscopy can be considered not to be mandatory during fURS.

**Abbreviations CIRF:**

clinically insignificant residual fragment; CT: computed tomography; EAU: European Association of Urology; (f)fURS: (fluoroless) flexible ureteroscopy; FT: fluoroscopy time; KUB: plain abdominal radiograph of the kidneys, ureters and bladder; mSv: millisievert; PCNL: percutaneous nephrolithotomy; pps: pulse-per-second; rem: roentgen equivalent man; PUJ: pelvi-ureteric junction; SFR: stone-free rate

## Introduction

Flexible ureteroscopy (fURS) is a successful treatment method for renal and upper ureteric stones. Over the years, this method has been more popular with urologists because of its less invasive nature compared to open surgery and percutaneous nephrolithotomy (PCNL). Additionally, technological and instrument developments have accelerated this process. According to the European Association of Urology (EAU) guidelines, fURS is an alternative first-line treatment modality to shockwave lithotripsy (SWL) for small renal stones (< 10 and 10–20 mm, suitable lower renal pole stones) and a second-line treatment modality for large renal stones (> 20 mm) [1].

Fluoroscopic imaging is required in conventional URS for stone imaging, determination of the renal anatomy, and patient safety [2,3]. The use of fluoroscopy in urological procedures has some risks such as malignancy inducement from radiation exposure of the patient, surgeon, and operation room staff [4,5]. There are some studies that have investigated the possibility of fluoroless URS during the treatment of stone disease [6–11]. In the present study, we investigated the feasibility and effectiveness of fluoroless (zero-dose) fURS (ffURS) for renal stone treatment in a large patient population.

## Patients and methods

The data of patients who underwent surgery for renal stones, between April 2013 and August 2018, were retrospectively evaluated. The data were screened from the patient’s records including surgical, medical and radiological history. Patients with stones in abnormal kidneys, pediatric patients (aged <18 years), simultaneously bilateral ureteric and renal stones, history of ureteric operation, history of surgical correction of PUJ obstruction, and stones in urinary diversions, were excluded from the study.

The patients with renal stone diameters of <20 mm and/or a failure of SWL were selected for ffURS. Informed consent was obtained from all patients included in the study. All patients were evaluated by urine analysis and culture, serum biochemistry, blood cell count, and coagulation tests. In the preoperative period, the imaging methods used in all patients included: CT urography or plain abdominal radiograph of the kidneys, ureters and bladder (KUB), and ultrasonography. The stone sizes were determined by the longest axis of the stones on CT or ultrasonography.

All operations were planned as ffURS. Patients in which fluoroscopy was required for any reasons were excluded from the study. These reasons included: i) stone migration and stone not found, ii) unsuccessful renal access, iii) suspected collecting system trauma, and iv) surgeon’s choice for any reason.

### FfURS technique

All procedures were performed with the patients under general or spinal anesthesia by experienced surgeons. Preoperative antibiotic therapy with third-generation cephalosporin was routinely administrated in the operation room. A C-arm fluoroscopic system (Siemens Healthiness, Erlangen, Germany) was placed in the operation room for use if necessary.

For routine fURS, a cystoscopy examination was performed routinely in all patients before the procedures. Then a semi-rigid URS (7.5 F) was routinely performed for ureteric dilatation, to check for any pathology in ureter and for any concomitant ureteric stones. Followed then by the insertion of a 0.089-cm (0.035-inch) guidewire into the renal pelvis, over which a ureteric access sheath (9.5/11.5 or 12/14 F, 35 or 45 cm, Cook Medical, Bloomington, IN, USA) was gently inserted to the renal pelvis. If the insertion of the access sheath or semi-rigid ureteroscope into the ureter was not possible, a JJ stent was inserted and the procedure was postponed until a second session. After the access sheath insertion, the pelvicalyceal system was monitored and the stones were located. A 7.5-F flexible ureteroscope (Flex X2, Karl Storz, Tuttlingen, Germany) was used for fURS. Then, fragmentation or dusting of the stone/stones was performed using a holmium laser generator (30-W; Dornier MedTech, Munich, Germany), until the stones were reduced in size or could be extracted with a basket catheter or completely dusted. Fragmented stones were extracted routinely with a nitinol basket catheter (Cook Medical). Stent placement was performed under endoscopic vision after the procedure and the stent was removed at 14–21 days postoperatively.

**Figure 1.**
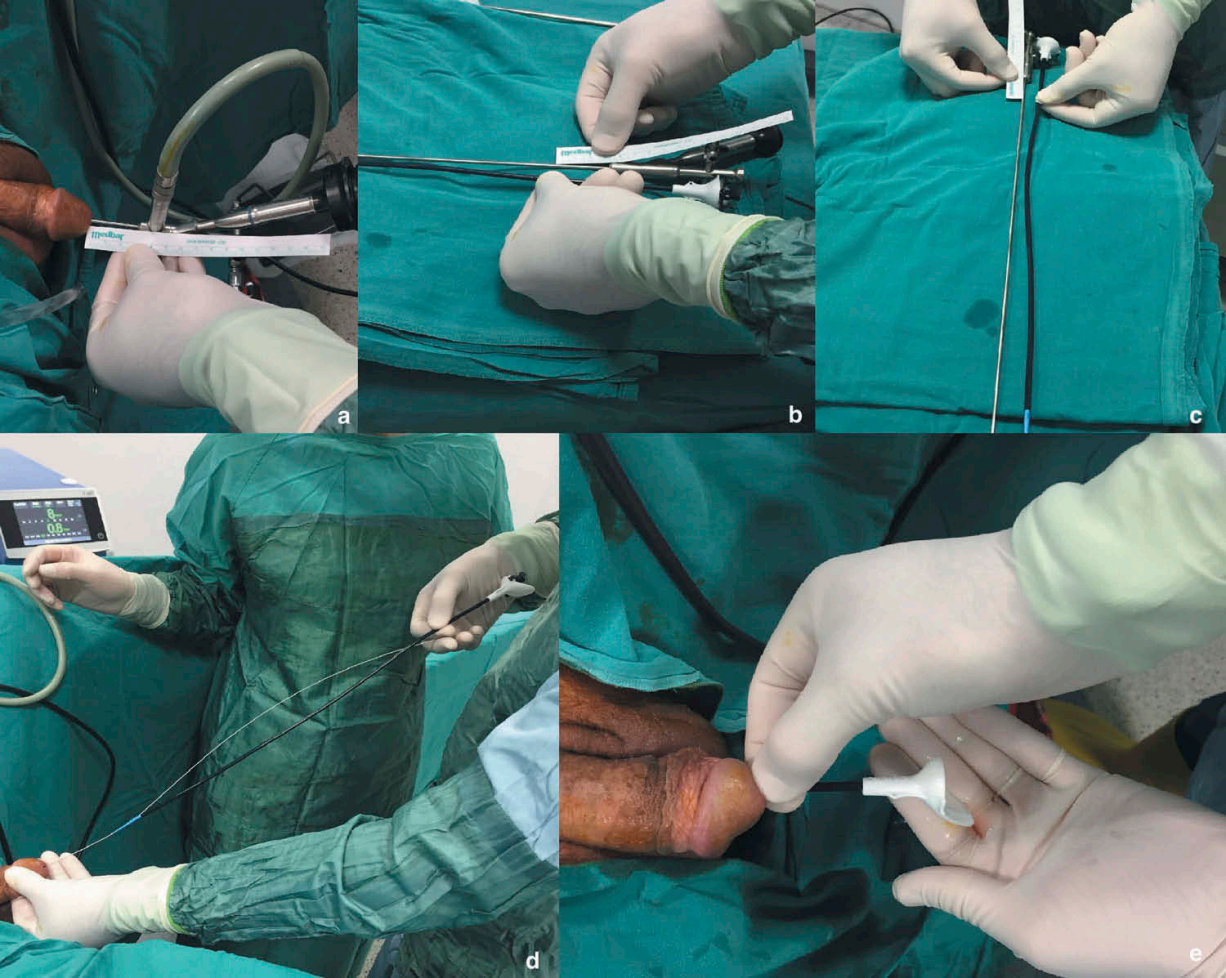
The step-by-step ffURS procedure: (a, b, c) The measurement of ureteric length for insertion of the ureteric access sheath after the semi-rigid URS. (d) Insertion of the ureteric access sheath over the guidewire. (e) Control of the ureteric access sheath placement with the urine drainage from the renal pelvis.

In our standard ffURS technique, fluoroscopy was not used. In this step-by-step technique all manipulations were performed with visual and tactile senses, as previously defined in pediatric patients [6] (Figure 1). The following steps were followed:

- A semi-rigid URS was performed over the safety guidewire under endoscopic vision.
- The size of ureter was calculated by semi-rigid URS for correct and safe ureteric access sheath insertion. When the tip of the ureteroscope was in the PUJ endoscopically, it was marked on the external part of the ureteroscope and the length between the tip of the ureteroscope and this marked line was measured.
- The ureteric access sheath was gently inserted over the guidewire into the renal pelvis as calculated before.
- The pelvicalyceal system was monitored and mapped.
- The stones were found.
- The stones were fragmented by holmium laser to small fragments, which could pass spontaneously.
- The larger stones fragments were removed with a nitinol basket.
- The ureteric access sheath was removed by checking the ureter under endoscopic vision with the flexible ureteroscope.
- A JJ stent was inserted using tactile senses and under endoscopic vision.

For all patients, the anesthesia time (preparation and wake-up of the patient) was subtracted from the operating time. Only surgical time was accepted as the operating time. For all the patients, demographic, perioperative, and postoperative parameters were evaluated.

### Follow-up

The results were classified as stone free, clinical insignificant residual fragments (CIRF), and unsuccessful. The absence of any residual stone was accepted as stone free. CIRF were defined as ≤4 mm, asymptomatic, non-obstructive stones on radiological imaging [12]. For all patients, the first follow-up evaluation was performed on the first day after surgery. On this day, a physical examination, pain status and urinary output were evaluated. The JJ stent was removed at 14–21 days postoperatively. All patients were controlled with ultrasonography or KUB before JJ stent removal. If there was a residual fragment, a second or third ffURS was performed.

At 3-months postoperatively, urine culture, creatinine level and low-dose CT were performed in all patients to evaluate the stone-free status. For the first year, the patients were evaluated every 3 months with urinary ultrasonography, plain radiography, and urinary pH, urine culture and creatinine level. They were then followed yearly. In the postoperative period, stone analysis and complete metabolic evaluation were performed in all patients.

### Statistical analysis

The Statistical Package for the Social Sciences (SPSS®), version 21.0 (SPSS Inc., Chicago IL, USA), was used to evaluate the patients’ data. The results were given as the mean ± standard deviation (SD). A *P* < 0.05 was considered statistically significant. Complications were graded using the Clavien–Dindo classification system [13]. Ureteric lacerations were classified as previously described in the literature [14].

## Results

The data of 744 patients who underwent surgery for renal stones were retrospectively evaluated. In all, 168 patients (22.5%) were excluded, with the remaining 576 patients planned for ffURS included in the study. Of these patients, ffURS was successfully achieved in 96.7% (557 patients). Of the 576 patients, 382 were male and 194 were female. The mean (SD) age of the patients was 44.7 (13.6) years and the mean (SD) stone size was 11.6 (5.2) mm. ffURS was not achieved in only 19 patients (3.3%); in seven of them the stone could not be found, and the JJ stent and ureteric access sheath could not be inserted in four and three patients, respectively. Additionally, in three patients the endoscopic view could not be effectively imaged due to hemorrhage. In two patients, the surgeon preferred to use fluoroscopy for checking the urinary system. There was no difference between 19 patients with unsuccessful ffURS and the remaining cases with successful ffURS in terms of demographic data, perioperative and postoperative outcomes, except for fluoroscopy times. In these 19 patients, the mean (SD) fluoroscopy time was 9.4 (3.6) s. The clinical data and patients’ characteristics are given in Table 1.

**Table 1.** Clinical data of the 576 patients.

Variable	Value
Age, years, mean (SD)	44.7 (13.6)
Stone size, cm, mean (SD)	11.6 (5.2)
Gender, *n* (%)	
Male	382 (66.3)
Female	194 (33.7)
Stone laterality, *n* (%)	
Left	309 (57.1)
Right	267 (42.9)
Stone localization, *n* (%)	
Upper calyx	74 (12.8)
Mid calyx	112 (19.4)
Lower calyx	167 (29.0)
Renal pelvis	153 (26.6)
Multiple	70 (12.2)
Presence of preoperative stent, *n* (%)	53 (9.2)
Preoperative hydronephrosis, *n* (%)	
No	428 (74.4)
Grade 1	95 (16.5)
Grade 2	40 (6.9)
Grade 3	13(2.2)
Achieved ffURS, n (%)	557 (96.7)

The majority of operations (98.2%) were performed under general anesthesia; spinal anesthesia was used in only 10 patients. The mean (SD) operating time was 39.4 (8.2) min. In all 557 patients with successful ffURS, at the end of the third month the success rate was (completely stone free) 83.3% (463 patients) and the CIRF rate was 7.3% (41 patients). Perioperative and postoperative outcomes are summarized in Table 2.

**Table 2.** Perioperative and postoperative outcomes of the 557 patients with successful ffURS.

Variable	Value
Operating time, min, mean (SD)	39.4 (8.2)
Hospitalization time, h, mean (SD)	21.4 (5.8)
SFR, *n* (%)	464 (83.3)
CIRF rate, *n* (%)	41 (7.3)
Second session ffURS rate, n (%)	32 (5.7)
Third session ffURS rate, n (%)	7 (1.2)
Ureteric access sheath insertion rate, *n* (%)	509 (91.3)
JJ-stent insertion rate, *n* (%)	523 (93.8)
Overall complication rate, *n* (%)	64 (11.8)
Renal colic	21 (3.7)
Fever	19 (3.4)
Urinary infection	11 (1.9)
Transient VUR	4 (0.7)
Paralytic ileus	3 (0.5)
Grade I ureteric laceration	4 (0.7)
Grade II ureteric laceration	2 (0.3)

Some patients required re-ffURS or additional treatments. A second and third session of ffURS was needed in 32 (5.7%) and seven (1.2%) patients, respectively. The second and third sessions of ffURS were performed due to residual or multiple or high-volume stones. In 12 (2.1%) patients, additional treatment procedures were performed. Of these, URS (fluoroless) for ureteric stones was performed in eight cases (1.4%), SWL was performed in two patients (0.3%), and PCNL was performed in two cases (0.3%).

During the study period, there were no major complications (Clavien–Dindo Grade III–IV). Of all patients, the overall complication rate was 11.8%. The most frequent complications were renal colic in 21 (3.7%), fever in 19 (3.4%), and urinary infection in 11 (1.9%) patients. Clavien–Dindo Grade I and II ureteric laceration was documented in four and two patients, respectively. Clavien–Dindo Grade I ureteric lacerations included simple ureteric erosion and Grade II ureteric lacerations included mucosal and smooth muscle injury. All ureteric lacerations were treated by JJ-stent insertion. For the 557 patients, all complications are listed in Table 2.

## Discussion

fURS is the most frequently performed endoscopic procedure for the treatment of renal and proximal ureteric stones of small/moderate size. Conventionally, this procedure requires the use of fluoroscopy during surgery for the safety of the operation process. However, most urologists mainly need fluoroscopic assistance during semi-rigid/rigid or fURS procedures. We know that fluoroscopy provides significant support during stone treatment, especially for fURS. It provides safe surgical stages such as localizing of the stones, identifying renal collecting system anatomy, performing retrograde pyelography, and insertion of the guidewire and JJ stent. However, the routine use of fluoroscopy is controversial.

It is well-known that medical radiation poses a potential risk of carcinogenesis in the patient, physician and other healthcare personnel. Thyroid, skin, extremity, and hematological malignancies can develop after chronic exposure to radiation [15–17]. Fluoroscopy is the main source of radiation exposure to the urologist. Not only the urologist, but also the patients and operation room staff are exposed to radiation during fluoroscopy. The main gauge of radiation exposure is fluoroscopy time (FT). In standard URS, the FT is variable. Hsi et al. [7], in their study including outcomes of nine URS series, reported a mean FT in URS of 144 s. Hellawell et al. [18] reported a mean (range) FT of 78 (6–414) s. Lipkin et al. [19] reported on 30 non-obese males who underwent URS; the mean FT was 47 s and the mean radiation dose was 0.31–7.17 millisieverts (mSv). According to these data, 1 pulse/s (pps) FT is approximately equal to a 0.01–0.02 mSv radiation dose. It is known that one chest X-ray is approximately equal to a 0.02 mSv radiation dose [20]. These data show that the ionized radiation scattered is as much as one chest X-ray per one pulse of fluoroscopy during URS, and all people (urologist, anesthetist, patient and others) are exposed to this ionized radiation dose. According to the National Council on Radiation Protection, the maximum yearly exposed radiation dose is 50 mSv (5000 roentgen equivalent man [millirem] or 5 rem) [5]. Per year, urologist can be safely exposed to 2500–5000 s of FT. It has been shown that this safety dose range is very low and that decreasing the radiation dose is essential for the safety of fURS.

Recently, urologists have investigated new modalities that reduce the FT or zero-dose techniques for the treatment of renal and ureteric stones. There are a limited number of published studies investigating low- or zero-dose fluoroscopy usage during fURS. We also previously investigated how to reduce the FT during fURS for renal stones with a limited number of patients in two centers [8] and described the ffURS technique for ureteric and renal stones in pediatric patients [6]. Hsi et al. [7] investigated FT during URS in their prospective study, which included 105 patients with ureteric and renal stones (162 renal units). In that study, a step-wise URS protocol was described and it was found that URS could be performed with the fluoroless protocol in 75% of the patients, whilst in 85% the FT was ≤ 2 s and in 95% the FT was ≤ 5 s. Yecies et al. [9] evaluated the effect of 1 pps fluoroscopy on FT and surgeon radiation exposure during URS. The authors included in the study a total of 84 patients and 70 underwent URS using continuous and 1 pps fluoroscopy, and found that 1 pps fluoroscopy is feasible, significantly reduces FT, and lowers surgeon radiation exposure by 64%.

In a retrospective comparative study including 100 patients with ureteric and renal stones, Olgin et al. [10] described a fluoroless URS technique in 50 of the patients. In that study, URS was performed using a step-by-step technique and it was found that the outcomes were similar in the two groups (conventional and fluoroless). The authors reported that the fluoroless technique was feasible and efficient for renal and ureteric stone treatment. In our present study, we included only patients with renal stones who underwent ffURS. Our present study patient numbers were greater than the Olgin et al. [10] and Hsi et al. [7] studies. Only 20 patients with renal stones were included in the Olgin et al. [10] study. In another study by Hein et al. [21], a novel method was described for ultra-low fluoroscopy usage in fURS. In that study, 174 procedures were assessed and they demonstrated that the exposure to ionising radiation could be significantly reduced using their protocol.

In another study by Senel et al. [11], the authors investigated 350 patients who underwent retrograde intrarenal surgery for renal and upper ureteric stones. In that retrospectively designed study, two groups were compared for retrograde intrarenal surgery with and without fluoroscopy; with 255 patients in the without-fluoroscopy group. They concluded that there was no difference between the two groups in terms of stone-free rate (SFR) and complications. The authors concluded that ffURS was a feasible and safe technique.

Traditional URS has effective SFRs and a low/minor complication rate. In large series, the SFR is reported to be 84–91% [1,22–24]. Also, it is has been shown that the overall complication rate of fURS is 9–25% and generally most complications are minor [1,11,25,26]. In our present study, we found that the primary SFR was 83.3% and the CIRF rate was 7.3%. If we included the 41 patients with CIRF, then the overall SFR was 90.6%. The overall complication rate was 11.8% and all the complications were minor. According to our present results, the SFR and complication rate are comparable to published data. We think that ffURS is an effective treatment modality for renal stones and has a low complication rate. Looking at the published data, there are few studies on reducing fluoroscopy usage during fURS. In our present study, we assessed a ffURS protocol in a large number of patients and all the patients had renal stones. As far as we know, the present study of ffURS for renal stones is the largest to date. We found that the ffURS technique is efficient and feasible for renal stone treatment, and has a reasonable SFR and low complication rate similar to conventional retrograde ureteric surgery.

Although the present study was carried out in a large population, there are some shortcomings. The study is not prospective or randomized. We believe that prospective, randomized and controlled studies would contribute to the data in the literature.

In conclusion; fluoroscopy usage is essential during ureteric and renal stone treatment, but it is not mandatory in experienced centers. ffURS is a safe and effective technique. The outcomes of this technique are comparable to conventional fURS. We think that, during fURS for renal stones, fluoroscopy is not mandatory and it should not be routinely used; however, if it is necessary, it should be used judiciously. We expect this technique will be routinely used in the future.
